# Data on the push–out bond strength of three different root canal treatment sealers

**DOI:** 10.6026/97320630017067

**Published:** 2021-01-31

**Authors:** Deepak Kurup, Ajay Kumar Nagpal, Shashit Shetty, Tapan Kumar Mandal, Juhi Anand, Rajdeep Mitra

**Affiliations:** 1 Department of Conservative Dentistry & Endodontics, Hazribagh college of dental sciences & hospital, Jharkhand, India; 2 Department of Conservative Dentistry & Endodontics, Kanti Devi Dental College and Hospital, Mathura, India; 3 Department of Restorative Dental Science, College Of Dentistry, King Khalid University, Abha, Kingdom of Saudi Arabia

**Keywords:** AH Plus, Apexit Plus, MTA Fillapex, push-out bond strength, bond failures

## Abstract

It is of interest to document data on the push – out bond strength of three different root canal treatment sealers such as MTA Fillapex (MTA based), AH plus (Epoxy Resin based) and Apexit plus (Calcium hydroxide based). Forty-five freshly extracted human maxillary central incisors with closed apices were selected randomly. All the teeth were sectioned at cement-enamel junction using a diamond disc before starting the root canal preparation to obtain root length of 12 mm. All teeth were instrumented using ProTaper rotary instruments. 5.25% sodium hypochlorite was used for irrigation between instrumentation followed by 17% EDTA, and final rinse by saline. Obturation procedures were done using the gutta-percha single cone technique. 45 roots were randomly assigned to 3 groups of 15 for obturation with gutta-percha cones and 1 of the 3 sealers (n=15). Group 1 = MTA Fillapex sealer + gutta-percha: Group 2 = AH plus sealer + gutta-percha:Group 3 = Apexit plus sealer + gutta-percha. The roots were sectioned horizontally to its canal into 3 sections: Coronal, Mid-root and Apical-thirds using a precision cutting machine, with a thickness of 3 mm. The specimens were subjected to push-out test using a universal testing machine that carried a plunger. The loading speed was 1mm/min until the dislodgment of the material occurred. The independent t- test was used to compare the mean scores among the study groups. The level of significance was set at 5% for all tests. After the push-out bond strength test, each sample was evaluated under stereomicroscope (40x) to determine the mode of failure and recorded as one of the following categories: adhesive, cohesive or mixed. The observations thus obtained were subjected to statistical analysis using Student - t test. AH Plus showed significantly higher values than MTA Fillapex and Apexit plus (p < 0.05). Amongst the push-out bond strength AH Plus sealer showed significant difference from MTA Fillapex and Apexit plus groups. There was no significant difference between MTA Fillapex and Apexit plus however (p>0.05). Microscopic analysis displayed that the majority of the modes were cohesive failures for AH Plus, adhesive failures for MTA Fillapex and mixed failures for Apexit Plus. . Thus, AH Plus had the highest bond strength and MTA Fillapex had the lowest bond strength to root dentin. Mean push-out bond strength values were ranked as follows; AH Plus >Apexit Plus > MTA Fillapex. Microscopic analysis displayed that the majority of the modes were cohesive failures of AH Plus, adhesive failures for MTA Fillapex and mixed failures for Apexit Plus.

## Background:

The purpose of the root canal sealer is to fill the interface between the core material and the dentin wall, to obtain a hermetic apical seal [1]. An ideal root canal sealer should dimensionally stable, a slow set to ensure sufficient working time, insoluble in tissue fluids, adequate adhesion with canal walls, biocompatible and provide an excellent seal when set [2]. Sealers based on dentin adhesion to seal the root canal more effectively. MTA Fillapex (Angelus, Londrina PR, Brazil) is a root canal sealer with high sealing capacity, which promotes regeneration of cementum. AH Plus is an epoxy resin sealer which is having excellent sealing properties and considered to be a gold standard [3]. The interfacial strength and dislocation resistance between the root filling material and the intra radicular dentin have been evaluated using thin-slice push out tests [4]. Therefore, it is of interest to document data on the push – out bond strength of three different root canal treatment sealers such as MTA Fillapex (MTA based), AH plus (Epoxy Resin based) and Apexit plus (Calcium hydroxide based).

## Material and Methods:

### Dataset:

Forty-five freshly extracted human maxillary central incisors with completely formed apices and straight canals were selected and stored in normal saline until use. The external root surfaces were cleaned of debris and hard deposits using the ultrasonic scaler, and sectioned at CEJ using a diamond disc to obtain roots of 12 mm in length.

### Model analysis:

The working length was determined such that, a size 15 K-file (DentsplyMaillefer, Ballaigues, Switzerland) was inserted into the root canal until it could be visualized at the apical foramen and then subtracting 1mm from this length. All teeth were instrumented using ProTaper rotary instruments (Dentsply- Maillefer, Ballaigues, Switzerland), attached with X-Smart endomotor (Dentsply- Maillefer, Ballaigues, Switzerland) at 300-rpm tillmaster apical file F3. 5.25% sodium hypochlorite (Chemident) was used for irrigation between instrumentation followed by 17% EDTA (Glyde, Dentsply, N.A.), and final rinse by saline.

### Obturation procedures were performed Background:

The purpose of the root canal s Background:

The purpose of the root canal sealer is to fill the interface between the core material and the dentin wall, to obtain a hermetic apical seal1. An ideal root canal sealer should dimensionally stable, a slow set to ensure sufficient working time, insoluble in tissue fluids, adequate adhesion with canal walls, biocompatible and provide an excellent seal when set [2]. Sealers based on dentin adhesion to seal the root canal more effectively. MTA Fillapex (Angelus, Londrina PR, Brazil) is a root canal sealer with high sealing capacity, which promotes regeneration of cementum. AH Plus is an epoxy resin sealer which is having excellent sealing properties and considered to be a gold standard [3]. The interfacial strength and dislocation resistance between the root filling material and the intra radicular dentin have been evaluated using thin-slice push out tests [4]. Therefore, it is of interest to document data on the push - out bond strength of three different root canal treatment sealers such as MTA Fillapex (MTA based), AH plus (Epoxy Resin based) and Apexit plus (Calcium hydroxide based). Ealer is to fill the interface between the core material and the dentin wall, to obtain a hermetic apical seal1. An ideal root canal sealer should dimensionally stable, a slow set to ensure sufficient working time, insoluble in tissue fluids, adequate adhesion with canal walls, biocompatible and provide an excellent seal when set [2]. Sealers based on dentin adhesion to seal the root canal more effectively. MTA Fillapex (Angelus, Londrina PR, Brazil) is a root canal sealer with high sealing capacity, which promotes regeneration of cementum. AH Plus is an epoxy resin sealer which is having excellent sealing properties and considered to be a gold standard [3]. The interfacial strength and dislocation resistance between the root filling material and the intra radicular dentin have been evaluated using thin-slice push out tests [4]. Therefore, it is of interest to document data on the push – out bond strength of three different root canal treatment sealers such as MTA Fillapex (MTA based), AH plus (Epoxy Resin based) and Apexit plus (Calcium hydroxide based). Using the F3 Protaper gutta-percha single cone technique and sealer. The 45 roots were randomly assigned to 3 groups of 15 each depending on the sealer used (n=15).

1) Group 1 = MTA Fillapex sealer 

2) Group 2 = AH plus sealer 

3) Group 3 = Apexit plus sealer 

## Results:

**Figure 1:**
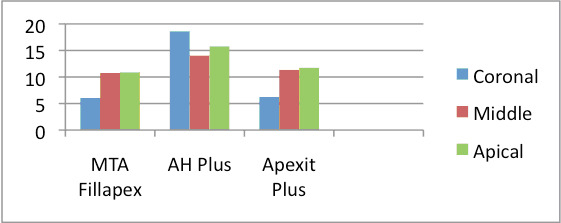
Mean of the push-out strength (in MPa) in the coronal, middle and apical third of each group.

AH Plus showed significantly higher bond strength than MTA Fillapex and Apexitplus (p < 0.05). There was no significant difference between group 1 and group 3 (p>0.05). AH Plus had the highest bond strength and MTA Fill apex had the lowest bond strength to root dentin (Table 1 - see PDF). Amongst the push-out bond strength of the coronal, middle and apical third specimens of the groups, there was no significant difference between MTA Fillapex and Apexit plus groups, however there was significant difference between AH Plus and the other two groups (Figure 1). Microscopic analysis displayed that the majority of the failures were cohesive for AH Plus, adhesive for MTA Fillapex and mixed for Apexit Plus (Table 2 - see PDF).

## Discussion:

The success of the root canal treatment depends mainly on the thorough debridement of the root canal system, the elimination of pathogenic organisms and finally the complete sealing of the canal space, which prevents ingress of bacteria from the oral environment into the root canal and spread to the periapical tissues. The properties required for this function include adaptation and adhesion of the filling material to the root canal dentinal wall, because gutta-percha does not directly bond to the dentin surface. Ideally, the sealer should be capable of producing a bond between core material and dentin wall [5]. The base material of MTA Fillapex is mineral trioxide aggregate (MTA); has a good sealing property, a bactericidal effect and is biocompatible [3]. MTA Fillapex is two-paste MTA-based salicylate resin root canal sealer. It has easy delivery method with good handling properties and an efficient setting time, which results in less wastage of material. One tube of MTA Fillapex formula contains 13.2% MTA. The other tube of MTA Fillapex contains biologically compatible salicylate resin (1,3 butylene glycol disalicylate resin), which is tissue friendly and therefore a better choice over epoxy-based resins, which have been shown to have mutagenic and more cytotoxic effects. MTA Fillapex two pastes combine in a homogenous mix to form a rigid, but semi permeable structure with excess MTA dispersed throughout [6]. The Dycal (Dentsply-Caulk, Milford, DE) is a calcium hydroxide containing pulp-capping agent, became popular as a sealer among some clinicians in late 1970s. Shortly afterward, root canal sealers based on calcium hydroxide became available in the market. Because calcium hydroxide-containing sealers have been in use over a quarter of a century and remain popular, a literature review on these materials focusing on their physical and biological properties is timely [7]. In the present study, we found that AH plus sealer was superior to MTA Fillapex and Apexit plus in terms of bond strength. The push out strength of the AH Plus sealer in the coronal, middle and apical third was found to be statistically significant when compared with the push out strengths of MTA Fillapex (tvalue: 12.296,2.420,2.513) and ApexitPlus (t-value: 12.062, 2.136,2.115). U. Salzet al (2009) concluded that Apexit Plus had a better sealing ability in comparison with AH Plus [1] because they found that AH Plus (0.3% solubility) had slightly lower solubility than Apexit Plus (0.5% solubility). McMichen F et al (2003) also investigated the solubility of Apexitplus and noted its very high solubility compared with AH Plus and Tubliseal [8]. We may therefore hypothesize that this significant difference in solubility between Apexit Plus and AH Plus may be responsible for the setting reaction of AH Plus to be more consistent than that of Apexit Plus which may further contribute to its increased bond strength. Moreover, AH Plus has very low shrinkage while setting and its long-term dimensional stability may also contribute to its increased bond strength. Numerous investigations have shown that the resin based sealer AH plus has higher bond strength than most other sealers. Harold H. Messer et al (2007) also evaluated the push-out bond strength of the dentin sealer interface with and without a main core for AH Plus, EndoREZ and Resilon. They observed that overall; the epoxy resin based sealer AH Plus provided the highest push-out bond strengths [5] which is similar to our study. The epoxy resin based sealer (AH Plus) has good penetration in micro irregularities due to high creep capacity and high polymerization time. These properties facilitate the interlocking between sealer and dentin, which allied to the cohesion among molecules, promotes larger adhesion and higher resistance to the sealer dislodgement from dentin surface [9]. We used single-cone obturation technique in the present study which can also result in a greater sealer thickness which in turn can influence the sealing ability of the root canal filling except when using AH Plus sealer. This phenomenon, along with its inherent volumetric expansion, may have contributed to the superior bond strength found with this epoxy resin-based sealer in the present study [10]. The results of our study confirmed the observations made by previous researchers about the calcium hydroxide based sealers. The major concern of sealers containing calcium hydroxide is that they might dissolve; leaving behind obturation voids [11], which will result in leakage. Calcium hydroxide based sealers also have poor cohesive strength as well as there is no objective proof that calcium hydroxide sealer provides any advantage for root canal obturation or has any of the desirable biological effect of calcium hydroxide paste [7]. In our study, we also observed that the results came out to be statistically in significant (t-value: 0.448, 0.407, 0.379; p>0.05) on comparing the push out strengths in the coronal, middle and apical thirds between MTA Fillapex and ApexitPlus.

## Conclusion

It is of interest to document data on the push - out bond strength of three different root canal treatment sealers such as MTA Fillapex (MTA based), AH plus (Epoxy Resin based) and Apexit plus (Calcium hydroxide based). Data shows that AH Plus had the highest bond strength and MTA Fillapex had the lowest bond strength to root dentin. Mean push-out bond strength values were ranked as follows; AH Plus >Apexit Plus > MTA Fillapex. Microscopic analysis displayed that the majority of the modes were cohesive failures of AH Plus, adhesive failures for MTA Fillapex and mixed failures for Apexit Plus.

## References

[1] Salz U (2009). International Endodontic Journal.

[2] Hui-min Zhou (2013). JOE.

[3] Sonmez IS (2013). Eur Arch Paediatr Dent.

[4] B Sagsen (2011). International Endodontic Journal.

[5] Jainaen AJ (2007). International Endodontic Journal.

[6] Milton Carlos Kuga (2011). RSBO.

[7] Shalin Desai, Nicholas Chandler (2009). J Endod.

[8] McMichen F (2003). IntEndod J.

[9] Nagpal A (2012). The Journal of Contemporary Dental Practice.

[10] Nagas E (2011). International Endodontic Journal.

[11] E Schafer T (2003). International Endodontic Journal.

